# Association between alcohol and tobacco use and primary open-angle glaucoma: the Eyes of Africa study

**DOI:** 10.1186/s40662-026-00515-y

**Published:** 2026-10-10

**Authors:** Oluwatobi J. Hussain, Olusola Olawoye, Kelsey V. Stuart, Tarela Sarimiye, Chimdi Chuka-Okosa, Ugbede Idakwo, Naa Naamuah Tagoe, Kwesi Nyan Amissah-Arthur, Kuburat O. Adenrele, Nkiru Kizor-Akaraiwe, Chinedu Anthony Okeke, Akinyemi O. Adedeji, Bola Adekoya, Aderonke Baiyeroju, Onoja M. Akpa, Oluwole Majekodunmi, Nkechi Uche, Bernice Adegbehingbe, Adeola Onakoya, Oyetunde E. Oyedepo, Chimdia Ogbonnaya, Bolajoko Abidemi Adewara, Joseph Matiya Msosavict, Akosua Badu Mensa-Bonsu, Kwaku Oppong Kwarteng, Yakubu Seidu Adam, Kwame Okyere Osei, Edem Lodoh, Benjamin Abaidoo, Raymond Toseafa, Suhanyah Okeke, Obinna Shiweobi, Steve Adebusoye, Kayode Fowobaje, Benedict Gboyega Ajayi, Adeyinka Falusi, Oluwafumbi Adeboye, Promise Okoroafor, Ifeyinwa Obianwu, Caroline Adeoti, Adedapo Adejuwon, Nathan Congdon, Anthony P. Khawaja, Michael A. Hauser, Tony Realini, Adeyinka Ashaye

**Affiliations:** 1 Department of Ophthalmology, College of Medicine, University of Ibadan, Ibadan, Nigeria; 2 Department of Ophthalmology, University College Hospital, Ibadan, Nigeria; 3 NIHR Biomedical Research Centre, Moorfields Eye Hospital NHS Foundation Trust and UCL Institute of Ophthalmology, London, UK; 4 Department of Ophthalmology, University of Nigeria Teaching Hospital, Ituku-Ozalla, Enugu, Nigeria; 5 ECWA Eye Hospital, Kano, Nigeria; 6 Lions International Eye Centre, Korle Bu Teaching Hospital, Accra, Ghana; 7 Ophthalmology Unit, Department of Surgery, University of Ghana Medical School, Accra, Ghana; 8 Ancilla Catholic Hospital Eye Centre, Lagos, Nigeria; 9 Department of Ophthalmology, College of Medicine, Enugu State University of Technology, Enugu, Nigeria; 10 The Eye Specialists Hospital (TESH), Enugu, Nigeria; 11 Department of Ophthalmology, Nigerian Navy Reference Hospital, Ojo, Lagos State Nigeria; 12 Department of Ophthalmology, Federal Medical Centre, Asaba, Nigeria; 13 Department of Ophthalmology, Lagos State University Teaching Hospital, Ikeja, Lagos Nigeria; 14 Division of Epidemiology, Biostatistics and Environmental Health, School of Public Health, University of Memphis, Memphis, TN USA; 15 Department of Ophthalmology, Obafemi Awolowo University/Teaching Hospitals Complex, Ile-Ife, Nigeria; 16 Lagos University Teaching Hospital Idi Araba/College of Medicine University of Lagos, Lagos, Nigeria; 17 Guinness Eye Centre, Lagos University Teaching Hospital Idi Araba, Lagos, Nigeria; 18 Alex Ekwueme Federal University Teaching Hospital, Abakaliki, Nigeria; 19 The Eyes for Africa Child Legacy Hospital, Lilongwe, Malawi; 20 Greater Accra Regional Hospital, Accra, Ghana; 21 Cocoa Clinic Eye Centre, Accra, Ghana; 22 Department of Epidemiology and Medical Statistics, University of Ibadan, Ibadan, Nigeria; 23 Eleta Eye Institute, Ibadan, Nigeria; 24 Trustee – Sickle Cell Hope Alive Foundation (SCHAF), Ibadan, Nigeria; 25 Department of Ophthalmology, Osun State University Teaching Hospital, Osogbo, Nigeria; 26 Centre for Public Health, School of Medicine, Dentistry and Biomedical Sciences, Queen’s University Belfast, Belfast, UK; 27 Orbis International, New York, NY USA; 28 Zhongshan Ophthalmic Centre, Sun Yat-Sen University, Guangzhou, China; 29 Department of Ophthalmology, Duke University, Durham, NC USA; 30 Department of Medicine, Duke University, Durham, NC USA; 31 Department of Ophthalmology and Visual Sciences, West Virginia University, Morgantown, WV USA; 32 MRC Epidemiology Unit, University of Cambridge, Cambridge, UK; 33 Rothschild Foundation Hospital, Paris, France; 34 Department of Ophthalmology and Visual Sciences, The Chinese University of Hong Kong, Hong Kong, China

**Keywords:** Primary open-angle glaucoma, Substance use, Alcohol consumption, Tobacco smoking, Africa, Cross-sectional study

## Abstract

**Background:**

Primary open-angle glaucoma (POAG) is the most prevalent glaucoma subtype globally and has disproportionately high rates in Africa. While smoking and alcohol consumption are established risk factors for several ocular conditions, their associations with POAG remain unclear. This study examined substance use patterns among individuals with and without POAG in sub-Saharan Africa.

**Methods:**

This cross-sectional study included 9,418 participants aged ≥ 40 years (5,189 POAG cases and 4,229 controls) from the Eyes of Africa: Genetics of Blindness study conducted across Nigeria, Ghana, and Malawi between 2017 and 2023. Comprehensive ocular examinations were performed, and POAG was diagnosed according to the International Society of Geographical and Epidemiologic Ophthalmology criteria. Sociodemographic data and detailed information on the use of substances including alcohol, tobacco, pharmaceutical substances, and illicit drugs, were collected through structured interviews. Chi-squared tests were used to examine the associations between variables, and multivariable logistic regression was used to estimate the odds ratios (ORs) and 95% confidence intervals (CIs) for substance use and POAG status.

**Results:**

Alcohol consumption and tobacco smoking were strongly and independently associated with POAG (*P* < 0.001). Former (OR = 1.49; 95% CI: 1.33–1.66; *P* < 0.001) and current drinkers (OR = 1.15; 95% CI: 1.00–1.31; *P* = 0.04) had higher odds than never-drinkers. Lifetime heavy episodic drinking (> 2 drinks per occasion) was associated with 40% higher odds of POAG (95% CI: 1.20–1.65; *P* < 0.001) than having ≤ 2 drinks. Tobacco use was also independently associated with POAG among both former (OR = 1.38; 95% CI: 1.13–1.68; *P* = 0.002) and current smokers (OR = 1.46; 95% CI: 1.12–1.91; *P* = 0.005). In addition, older age, male sex, higher educational level, and unemployment (all *P* < 0.001) were independently associated with POAG.

**Conclusion:**

Cigarette smoking and alcohol use are significantly associated with increased risk of POAG in this African population, representing potentially modifiable risk factors. Confirmatory longitudinal studies are needed to establish causality. If validated, public health strategies for POAG prevention in Africa should incorporate substance use reduction as a key component of primary prevention.

**Supplementary Information:**

The online version contains supplementary material available at https://doi.org/10.1186/s40662-026-00515-y.

## Background

Glaucoma is a chronic progressive optic neuropathy that causes vision loss and irreversible blindness. Globally, the number of individuals with glaucoma aged over 40 years is estimated at 64.3 million, projected to be 111.8 million by 2040 [1]. Primary open-angle glaucoma (POAG) is the most prevalent subtype of glaucoma, accounting for approximately 74% of cases globally [2]. It is also the most common form among individuals in Africa and those of African descent [3, 4], with a reported prevalence of 4.20% [1]. Currently, an estimated 8.7 million people aged 40 to 80 years live with POAG in Africa, which is projected to nearly double to 16.2 million by 2040 [1].

Many risk factors have been associated with POAG, including older age, family history of glaucoma, African ancestry, and increased intraocular pressure (IOP) [5]. The primary modifiable cause of POAG development and progression is elevated IOP [6]. However, not all patients with ocular hypertension will develop POAG, and POAG can develop even when the IOP appears to be normal [7]. This suggests that additional factors beyond IOP contribute to POAG pathogenesis.

Environmental factors such as smoking and alcohol consumption may influence oxidative stress and vascular perfusion to the optic nerve [8]. Anatomic variations in the optic nerve head and lamina cribrosa structure can affect susceptibility to glaucomatous damage [9]. Physiological factors, including systemic blood pressure, nocturnal hypotension, and ocular perfusion pressure, may also play important roles in disease development [10]. Genetic predisposition further modulates individual risk through inherited variations affecting trabecular meshwork function, retinal ganglion cell survival, and optic nerve vulnerability [11]. These data collectively demonstrate that POAG is a complex interplay of genetic, environmental, anatomic, and physiological variables [12].

Smoking is a prevalent and modifiable risk factor for numerous ocular conditions, including cataracts and age-related macular degeneration [13]. However, compared with other diseases like cancer and cardiovascular disease, the impact of smoking on eye diseases has been less well studied [14]. The epidemiological evidence linking tobacco smoking to POAG is inconsistent. Several biologically plausible pathways have been proposed, including vasoconstriction of the episcleral veins, impairment of vasoactive mediators such as nitric oxide and endothelin-1, and oxidative damage to the trabecular meshwork and retinal ganglion cells. However, two recent large-scale systematic reviews and meta-analyses that collectively encompassed hundreds of thousands of participants found no statistically significant association between smoking, whether current, former, or any, and POAG incidence [15, 16]. These null findings highlight the need for further investigations across diverse populations, particularly those of African ancestry, among whom the POAG burden is disproportionately high.

Alcohol consumption is a substantial cause of global morbidity and mortality and a risk factor for several chronic diseases that affect different organ systems [17]. Alcoholic neuropathy is more common in heavy drinkers. It has been linked to chronic excessive alcohol intake through oxidative stress, nutritional deficiencies, free radical damage to neurons, and direct toxic repercussions [18]. Excessive consumption of alcohol may damage optic neurons through pathways akin to alcoholic neuropathy. According to recent studies, alcohol consumption is associated with increased IOP and the risk of developing POAG [19, 20]. A linear dose–response association between alcohol use and glaucoma-related outcomes (e.g., higher IOP, thinner retinal nerve fiber layer, and higher POAG prevalence) has also been reported [19]. These findings may indicate that non-drinkers or moderate drinkers are less likely to acquire POAG and have a better prognosis than heavy drinkers [21].

The lack of comprehensive studies on the relationship between substance use and POAG in Africa highlights a considerable gap in the current body of knowledge. Addressing this gap is imperative for providing a nuanced understanding of how lifestyle factors influence the prevalence and progression of POAG, ultimately contributing to improved health outcomes in those affected by this debilitating condition. This comparative analysis is essential for developing targeted public health interventions and informing clinical practices to mitigate the impact of POAG in African populations.

## Methods

This was a cross-sectional study of patients recruited in the Eyes of Africa: Genetics of Blindness (EOA: GOB) study. All EOA: GOB participants were recruited as a part of a multicenter study. The EOA study methodology and recruitment protocol have been described previously [22]. Briefly this was a cross-sectional study conducted at five main sites in sub-Saharan Africa, three sites in Nigeria, one in Ghana, and one in Malawi.

The study protocol was reviewed and approved by the University of Ibadan/University College Hospital Ethics Review Committee (UI/EC/17/0394) and the relevant ethics committees of participating institutions, (The Ghana Health Service Review Committee: GHS-ERC 011/12/20, Korle Bu Teaching Hospital Review Committee: KBTH-IRB/00033/2021, University of Nigeria Teaching Hospital Ethics Review Committee: UNTH/CSA/329/OL.5 IRB00002323). Written consent to participate was obtained from all potential participants in their local language before screening and any study-related interventions (including examination for eligibility). Eligible participants who wished to participate were enrolled in the study. The study used broad consent to enable the use of the samples and associated clinical data in other H3Africa-sponsored research projects [23].

A total of 9418 participants aged ≥ 40 years were consecutively recruited for the EOA: GOB study from 2017 to 2023 through a total sampling technique. Patients were diagnosed with POAG, defined as the presence of glaucomatous optic neuropathy with cupping, pallor, thinning, or loss of the neuroretinal rim detected through optic nerve head examination and/or optical coherence tomography of the retinal nerve fiber layer, with characteristic visual field loss, open angles on gonioscopy, and no other ocular abnormalities to account for these changes. The International Society of Geographical and Epidemiological Ophthalmology criteria [24] were used to define glaucoma as detailed in our methodology, which uses three levels of evidence and has been described in our previous publication [22]. When a visual field test could not be performed due to very poor vision, diagnosis was made based on Level 2 evidence, specifically, advanced structural damage to the optic disc in the absence of confirmed visual field loss. The controls had IOP < 22 mmHg and no evidence of glaucomatous optic neuropathy or other clinical evidence of glaucoma of any type. The patients were predominantly enrolled (98.6%) from glaucoma clinics, and only a small proportion (1.4%) was identified from community outreach programs. The controls were recruited from cataract clinics and general outpatient geriatric clinics (93.1%) and from community outreach programs (6.9%). Consecutive eligible participants were invited to participate in the study to minimize selection bias.

Basic demographic information, including age, sex, height, weight, educational level, employment status, country of birth, ethnicity, and native language, was collected. Information regarding medication history, substance use, smoking status, and alcohol consumption was also obtained. Based on alcohol consumption, participants were categorized into never drinkers: never had at least one drink of alcohol excluding small tastes or sips; former drinkers: not drank alcohol in the last 30 days preceding the interview; and current drinkers: those who had had at least one drink in the last 30 days preceding the interview. Participants were also categorized as former smokers (more than 100 cigarettes over their lifetime but not in the last 30 days preceding the interview), current smokers (smoked tobacco in the last 30 days preceding the interview), or nonsmokers (never smoked up to 100 cigarettes in their lifetime). Medications included sedatives, tranquillizers, painkillers, and stimulants.

All the study participants underwent comprehensive ocular examinations. IOP was measured in both eyes using Goldmann applanation tonometry. Gonioscopy was performed on all participants, and the angles were graded according to Shaffer’s grading system. Only patients with open angles were included in this study. All eyes with open angles were dilated after gonioscopy. The optic disc was examined using a 78-D lens and a 10× eyepiece of a slit lamp for stereoscopic evaluation of the optic disc.

All analyses were performed using SPSS version 23.0. Chi-squared tests were used to examine the associations between sociodemographic variables, substance use (alcohol, tobacco, and other substances), and POAG status. To identify independent associations between substance use and POAG status, a multivariable logistic regression analysis was conducted. Variables showing significant associations with POAG in the bivariate chi-squared tests (*P* < 0.05) were entered into the initial logistic regression model. Backward elimination was performed using the Wald statistic, with variables sequentially removed if *P* > 0.10. Variables were retained in the final model if they achieved statistical significance (*P* < 0.05) or caused > 10% change in the odds ratios (ORs) of the key predictors, indicating confounding factors. Model fit was assessed using the Hosmer–Lemeshow goodness-of-fit test. The final significance level was set at α = 0.05, and ORs with 95% confidence intervals (CIs) were calculated.

An additional sensitivity analysis was conducted by repeating the multivariable model after including body-mass index (BMI) to assess whether the adjustment for adiposity altered the observed associations.

## Results

### Sociodemographic characteristics

A total of 9,418 participants, comprising 5,189 individuals with POAG and 4,229 healthy controls, were enrolled in this study. Table 1 shows the sociodemographic characteristics of the study population. Sex distribution differed significantly between the groups (*P* < 0.001), with a higher proportion of males in the POAG group (57.4%) than in the control group (45.2%). The age distribution revealed that participants aged 60–79 years represented the largest proportion in both groups, accounting for 45.1% of those with POAG and 41.9% of the controls. Notably, individuals aged ≥ 80 years were more prevalent in the POAG group (29.1%) than in the control group (21.7%), with the overall age distribution showing significant differences between the groups (*P* < 0.001).

**Table 1:** Sociodemographic characteristics of the participants (n = 9418)

Variable	Total	POAG (%)	Control (%)	*P* value
Sex				
Female	4529	2211 (42.6)	2318 (54.8)	< 0.001*
Male	4889	2978 (57.4)	1911 (45.2)	
Age (years)				
40–59	2882	1341 (25.8)	1541 (36.4)	< 0.001*
60–79	4110	2338 (45.1)	1772 (41.9)	
≥ 80	2426	1510 (29.1)	916 (21.7)	
Educational level (n = 9412)				
Less than secondary education	5288	2812 (54.2)	2476 (58.7)	< 0.001*
Secondary education and higher	4118	2374 (45.18)	1744 (41.3)	
Employment status				
Employed	5033	2568 (49.5)	2465 (58.3)	< 0.001*
Unemployed	4385	2621 (50.5)	1764 (41.7)	
Country of birth				
Nigeria	8761	4858 (93.6)	3903 (92.3)	0.012*
Non-Nigeria	657	331 (6.4)	326 (7.7)	
Ethnic affiliation				
Yoruba	4374	2448 (47.2)	1926 (45.5)	0.110
Non-Yoruba	5044	2741 (52.8)	2303 (54.5)	

*POAG* = primary open-angle glaucoma

^*^ Indicate statistically significant

### Prevalence of substance use

Table 2 presents the prevalence of alcohol and tobacco use patterns among the participants with and without POAG. Regarding alcohol consumption, a statistically significant difference was observed between the two groups (*P* < 0.001). In the POAG group, 56.4% reported never consuming alcohol compared with 65.0% of the controls. Former alcohol drinkers constituted a substantially larger proportion of cases (28.1%) than controls (20.5%), whereas current drinkers comprised similar proportions in both groups (15.5% vs. 14.5%, respectively). Similarly, tobacco smoking was significantly associated with POAG (*P* < 0.001). The proportion of participants who had never smoked was lower among the POAG cases (87.8%) than among the controls (93.4%). Former smokers accounted for 7.9% of cases compared with 4.3% of controls, and current smokers accounted for 4.3% of cases compared with 2.3% of controls.

**Table 2:** Prevalence of alcohol and tobacco consumption among individuals diagnosed with primary open-angle glaucoma (POAG) and those without the condition in Africa

Variables	POAG (%)	Controls (%)	χ^2^	*P* value
Alcohol (n = 9335)				
Never drank	2903 (56.4)	2721 (65.0)	83.19	< 0.001*
Former drinkers	1447 (28.1)	860 (20.5)		
Current drinkers	796 (15.5)	608 (14.5)		
Tobacco (n = 9280)				
Never smoked	4510 (87.8)	3874 (93.4)	82.28	< 0.001*
Former smokers	406 (7.9)	177 (4.3)		
Current smokers	218 (4.3)	95 (2.3)		
Sensitivity analysis				
Alcohol (n = 6745)				
Never drank	2855 (80.8)	2674 (83.3)	6.782	0.009*
Current drinkers	678 (19.2)	538 (16.7)		
Tobacco (n = 6745)				
Never smoked	3416 (96.7)	3154 (98.2)	15.097	< 0.001*
Current smokers	117 (3.3)	58 (1.8)		

^*^Indicate statistically significant

Supplementary Table 1 presents the prevalence of substance use among the participants with and without POAG. Among the pharmaceutical substances, painkiller use was significantly higher in the control group (74.0%) than in the POAG group (68.7%, *P* < 0.001), and tranquillizer use was also higher in the control group (4.2%) than in the POAG group (2.6%, *P* < 0.001). Sedative use was comparable between the groups, with approximately one-third of the participants in both the POAG (33.7%) and control groups (34.9%) reporting having used sedatives (*P* = 0.21). Stimulant use was relatively low in both groups, with no significant difference observed (5.2% in POAG vs. 4.5% in controls, *P* = 0.132).

Marijuana use (including hash, hemp concentrate, or grass) was significantly more prevalent among individuals with POAG (1.8%) than among controls (1.1%, *P* = 0.005). The use of other illicit drugs, including cocaine, crack-cocaine, hallucinogens, inhalants, and heroin, was rare in both groups, with a prevalence below 1%; no statistically significant differences were observed between the POAG and control participants.

### Patterns of alcohol and tobacco consumption

A more granular analysis of alcohol and tobacco use patterns among participants with a history of alcohol consumption is shown in Table 3. Among 3,708 participants who reported any alcohol use, a significant difference in drinking recency was observed (*P* < 0.001). Former drinkers who had not consumed alcohol within the preceding 30 days were more prevalent among POAG cases (64.5%) than controls (58.6%); however, current drinkers who had consumed alcohol within the last 30 days constituted a higher proportion of controls (41.4%) than cases (35.5%).

**Table 3:** Tobacco and alcohol use patterns between individuals with primary open-angle glaucoma (POAG) and those without in Africa

Variables	POAG (%)	Control (%)	χ^2^	*P* value
Alcohol use pattern (n = 3708)				
Former drinkers (not in the last 30 days)	1447 (64.5)	860 (58.6)	13.26	< 0.001*
Current drinkers (in the last 30 days)	796 (35.5)	608 (41.4)		
Average drinks/month in the last 30 days (n = 1404)				
≤ 60	775 (97.4)	590 (97.0)	0.133	0.720
> 60	21 (2.6)	18 (3.0)		
Largest amount of drink ever drunk in a single day (n = 3191)				
≤ 2	1311 (68.8)	971 (75.6)	17.33	< 0.001*
> 2	595 (31.2)	314 (24.4)		
Cigarette smoking pattern (n = 896)				
Former smokers (not in the last 30 days)	408 (65.3)	175 (64.6)	0.016	0.900
Current smokers (in the last 30 days)	217 (34.7)	96 (35.4)		
Average number of cigarettes smoked per day in the last 30 days (n = 313)				
≤ 5	153 (70.2)	69 (72.6)	0.192	0.660
> 5	65 (29.8)	26 (27.4)		

^*^Indicate statistically significant

When examining drinking intensity among current drinkers, the average number of drinks consumed in the preceding 30 days did not differ significantly between cases and controls (*P* = 0.72), with most of both groups consuming 60 or fewer drinks per month (97.4% of cases and 97.0% of controls). However, the largest amount of alcohol consumed in a single day differed significantly between groups (*P* < 0.001). Participants with POAG were more likely to report consuming larger quantities of alcohol in a single day, with 31.2% reporting more than two drinks as their largest amount ever consumed in a single day, compared with 24.4% of the controls (*P* < 0.001).

In contrast to alcohol consumption patterns, among the 896 participants with a smoking history, no significant difference was observed in smoking recency (*P* = 0.90); former smokers comprised 65.3% of POAG cases and 64.6% of controls, while current smokers accounted for 34.7% of cases and 35.4% of controls. Similarly, the average number of cigarettes smoked per day among current smokers did not differ significantly between the groups, with approximately three-quarters of smokers in both the POAG (77.5%) and control groups (79.0%) reporting the consumption of five or fewer cigarettes daily (*P* = 0.73).

### Factors associated with POAG

Multivariable logistic regression analysis, presented in Table 4, identified several significant associations between sociodemographic factors, substance use, and POAG. After adjustment for all covariates, males had 40% higher odds of POAG than females (OR = 1.40, 95% CI: 1.27–1.53, *P* < 0.001). Older age demonstrated a strong association with POAG, individuals aged 60–79 years had a 42% higher odds of POAG than those aged 40–59 years (OR = 1.42; 95% CI: 1.27–1.58; *P* < 0.001), whereas those aged ≥ 80 years had an 83% higher odds (OR = 1.83; 95% CI: 1.62–2.06; *P* <0.001) than those in the 40–59-year age group.

**Table 4:** Logistic regression of factors associated with POAG

Variable	AOR	95% CI	*P* value
Sex			
Female	Ref		
Male	1.40	1.27–1.53	< 0.001*
Age (years)			
40–59	Ref		
60–79	1.42	1.27–1.58	< 0.001*
≥ 80	1.83	1.62–2.06	< 0.001*
Educational level			
Less than secondary education	Ref		
Secondary education and higher	1.27	1.16–1.39	< 0.001*
Employment status			
Employed	Ref		
Unemployed	1.23	1.13–1.35	< 0.001*
Country of birth			
Nigeria	Ref		
Not in Nigeria	0.80	0.67–0.96	< 0.017*
Alcohol			
Never drank	Ref		
Former drinkers	1.49	1.33–1.66	< 0.001*
Current drinkers	1.15	1.00–1.31	0.044*
Tobacco			
Never smoked	Ref		
Former smokers	1.38	1.13–1.68	0.002*
Current smokers	1.46	1.12–1.91	0.005*
Tranquillizers			
Never used	Ref		
Ever used	0.62	0.49–0.80	< 0.001*
Painkillers			
Never used	Ref		
Ever used	0.75	0.68–0.82	< 0.001*
Marijuana			
Never used	Ref		
Ever used	1.18	0.80–1.74	0.41
Alcohol use pattern			
Not in the last 30 days	Ref		
In the last 30 days	1.29	1.12–1.47	< 0.001*
Largest amount of drink ever drunk in a single day			
≤ 2	Ref		
> 2	1.40	1.20–1.65	< 0.001*
Sensitivity analysis (restricted)			
Alcohol			
Never drank	Ref		
Current drinkers	0.90	0.78–1.04	0.166
Tobacco			
Never smoked	Ref		
Current smokers	1.62	1.16–2.27	0.005*

Variables in the multivariable regression model: sex, age, educational level, employment status, country of birth, alcohol use, tobacco use, tranquillizers, painkillers, marijuana, alcohol use pattern, and largest amount of drink ever consumed in a single day

*AOR* = adjusted odds ratio; *CI* = confidence interval; *Ref* = reference category

^*^Indicate statistically significant

Educational level and employment status were significantly associated with POAG. Individuals with secondary education or higher had 27% increased odds of POAG (OR = 1.27, 95% CI: 1.16–1.39, *P* < 0.001), while unemployment was associated with 23% higher odds (OR = 1.23, 95% CI: 1.13–1.35, *P* < 0.001). Interestingly, being born outside Nigeria was associated with 20% lower odds of POAG (OR = 0.80, 95% CI: 0.67–0.96, *P* < 0.001).

Regarding substance use, both former and current alcohol consumption were independently associated with elevated odds of POAG after full adjustment. Former drinkers exhibited the highest odds relative to never-drinkers (OR = 1.49; 95% CI: 1.33–1.66; *P* < 0.001), and current drinkers also showed a significant, albeit attenuated, association (OR = 1.15; 95% CI: 1.00–1.31; *P* = 0.04). The pattern of alcohol consumption further modulated this risk: those who had consumed alcohol in the last 30 days had 29% higher odds of POAG than those who had not (OR = 1.29; 95% CI: 1.12–1.47; *P* < 0.001). Moreover, lifetime heavy episodic drinking, which is defined as ever consuming more than two standard drinks in a single day in one’s lifetime, conferred a 40% increase in the odds of POAG compared with lighter drinking (OR = 1.40; 95% CI: 1.20–1.65; *P* < 0.001), suggesting that drinking intensity, rather than frequency alone, may be particularly important in the relationship between alcohol consumption and POAG.

Tobacco smoking was independently associated with POAG in both former (OR = 1.38; 95% CI: 1.13–1.68; *P* = 0.002) and current smokers (OR = 1.46; 95% CI: 1.12–1.91; *P* = 0.005) relative to those who had never smoked, with current smokers showing the highest odds. The use of tranquillizers and painkillers was associated with reduced odds of POAG, with adjusted ORs of 0.62 (95% CI: 0.49–0.80, *P* < 0.001) and 0.75 (95% CI: 0.68–0.82, *P* < 0.001), respectively. Although marijuana use was significantly more prevalent in POAG cases in the bivariate analysis, it did not reach statistical significance in the multivariable model (OR = 1.18, 95% CI: 0.80–1.74, *P* = 0.41).

An additional multivariable analysis incorporating BMI was performed to assess the robustness of the primary findings (Supplementary Table 2). Adjusting for BMI modestly attenuated the association between alcohol use and POAG, with current alcohol use no longer reaching statistical significance, whereas the associations with former alcohol use, heavy episodic drinking, tobacco smoking, and the principal sociodemographic factors remained broadly similar to those observed in the primary model.

In the sensitivity analysis restricted to current and never users of both alcohol and tobacco, current smoking remained independently associated with POAG (OR = 1.62; 95% CI: 1.16–2.27; *P* = 0.005). However, current alcohol consumption, was not significantly associated with POAG (OR = 0.90; 95% CI: 0.78–1.04; *P* = 0.166) and was excluded from the final model by the Backward Wald procedure.

## Discussion

To the best of our knowledge, this study represents the largest comparative analysis of substance use patterns among individuals with and without POAG in Africa, with 9,418 participants. This study provides important insights into the relationship between substance use patterns and POAG prevalence in an African population. Our findings demonstrated significant differences in substance use behaviors between individuals with POAG and controls, with alcohol consumption and tobacco smoking showing particularly strong associations with the disease. These results contribute to the growing body of evidence examining potentially modifiable risk factors for POAG in diverse populations, particularly in sub-Saharan Africa, where glaucoma remains the leading cause of irreversible blindness.

The male predominance in the POAG group (57.4%) aligns with prior African studies, with regression analysis showing 40% higher odds in men, consistent with reported male-to-female ratios of 1.2:1 to 1.8:1 [25]. While the neuroprotective effects of estrogen on retinal ganglion cells may partly explain the lower prevalence in women [26], gender-based barriers in sub-Saharan Africa, including limited autonomy, restricted mobility, and reduced healthcare access, may contribute to underdiagnosis among women [27].

The progressive increase in POAG odds with advancing age, reaching 83% higher odds among those aged ≥ 80 years, mirrors well-established glaucoma epidemiological patterns. This age-related gradient likely reflects cumulative structural and vascular changes in the optic nerve, age-related IOP increases, and reduced neuroprotective capacity [28]. Global data show that POAG incidence rose from 5.51 per 10,000 person-years in the 40–44-year age group to 64.36 per 10,000 person-years in the 75–79-year age group [29]. In Nigeria, late presentation, particularly among older individuals, remains a critical challenge [30]. This reinforces the need for age-stratified screening programs within primary eye care systems across Africa.

The positive association between higher educational attainment and POAG was intriguing and counterintuitive. Our findings suggest a 22% increase in odds among those with secondary education or higher, likely reflecting a selection bias, as individuals with higher education may have greater access to eye care services and are therefore more likely to be diagnosed with POAG. Similar patterns have been reported in urban African populations where educated individuals are more likely to utilize hospital-based eye care services [31]. The observation is also supported by clinical research, which shows that longer education significantly correlates with the development of myopia, which in turn independently doubles or triples the risk of developing POAG [32]. Alternatively, this association might relate to occupational factors, as higher education often correlates with sedentary work and prolonged near-work activities, which some studies have linked to POAG risk, although this remains controversial [33].

The association between alcohol use and POAG is one of the most striking findings of this study. Both former and current drinkers had significantly higher odds of POAG than never drinkers, even after full covariate adjustment. These findings are consistent with those of a systematic review and meta-analysis by Stuart et al. [20], who synthesized evidence from 34 studies and found that alcohol use was adversely associated with IOP and OAG risks.

Consuming more than two drinks as the largest amount ever consumed in a single day was associated with 40% increased odds of POAG, indicating that lifetime heavy episodic drinking may be particularly detrimental. These findings add to the complex literature on alcohol consumption and the risk of POAG. Stuart et al. found that moderate alcohol consumption showed no significant association with POAG, whereas heavy drinking was associated with increased risk [19]. Our results align with findings on adverse associations even at moderate consumption levels, including a large Korean cohort study reporting that alcohol consumption was associated with increased risk of normal-tension glaucoma with a dose–response relationship [11]. The mechanisms potentially linking alcohol to POAG include alcohol-induced elevations in IOP, effects on vascular regulation and ocular perfusion, oxidative stress, and disruption of neuroprotective pathways in the retina and optic nerve [19].

The intensity of alcohol consumption was also important. Active drinking during the preceding 30 days was independently associated with 29% higher odds of POAG, with former drinkers having the highest odds. This apparent paradox, where past rather than current drinking carries greater odds, has been observed in other large epidemiological studies [34, 35], and may reflect reverse causality. Individuals who have already developed POAG or IOP-related symptoms may have reduced their drinking as part of disease management or healthcare advice, whereas former drinkers represent those with a longer history of exposure before cessation [20].

Additionally, we evaluated whether adjustment for BMI influenced the observed associations by fitting an additional multivariable model (Supplementary Table 2). The inclusion of BMI modestly attenuated alcohol-related associations, particularly current alcohol use, whereas the associations with tobacco smoking and major sociodemographic variables remained broadly unchanged. BMI was not included in the primary model because adiposity may represent an intermediate metabolic factor or share behavioral and metabolic pathways linking alcohol consumption with POAG [36–38]. Given the cross-sectional nature of this study, these pathways cannot be distinguished, and the primary model was therefore retained to estimate the overall association between alcohol use, tobacco use, and POAG.

Our study found a significant independent association between tobacco smoking and POAG in both former and current smokers, with the highest odds observed among those who were still smokers at the time of the study. This represents a particularly important finding given the increasing prevalence of tobacco use in many African countries. This association was among the strongest associations observed in our multivariable model and remained significant after controlling for alcohol consumption and other confounders. Our findings are consistent with mounting evidence linking tobacco smoking to the risk of POAG. Pérez-de-Arcelus et al. found that current smoking was associated with 14% increased odds of POAG, with evidence of a dose–response relationship [14], and additional studies have reported even stronger associations, particularly in populations with high smoking prevalence [15]. The biological plausibility is supported by multiple mechanisms, including tobacco's vasoconstrictive effects, which lead to reduced ocular blood flow, increased oxidative stress causing damage to the trabecular meshwork and retinal ganglion cells, and potential direct toxic effects on the optic nerve [6].

However, among ever-smokers, neither the recency nor daily intensity of smoking significantly differentiated cases from controls, suggesting that the population-level association was driven by exposure prevalence rather than by a dose–response gradient. This is consistent with recent meta-analytic evidence reporting no clear dose–response relationship between cigarette consumption and POAG incidence [15]. Collectively, these findings imply that any tobacco exposure may confer a meaningful likelihood of POAG, reinforcing the importance of smoking cessation as a potential modifiable target in POAG prevention.

To address reverse causality, we repeated the multivariable analysis, restricting the sample to current and never users and excluding all former users of both substances. Current smoking remained independently associated with POAG, with the association strengthening across model steps, suggesting that this finding is robust and unlikely to reflect post-diagnosis behavioral changes. This is consistent with mechanistic evidence implicating nicotine-induced vasoconstriction, disruption of vasoactive mediators, and oxidative injury to the trabecular meshwork as plausible biological pathways [16, 39].

In contrast, alcohol consumption was not independently associated with POAG in this restricted analysis, suggesting that the finding of the primary analysis may reflect protopathic bias, whereby individuals reduce or cease drinking following diagnosis [40]. This is further supported by the paradoxical descriptive finding that current drinking was less prevalent among cases despite higher rates of heavy episodic drinking, a pattern more consistent with post-diagnosis behavioral modification than a true biological effect. Prospective studies are needed to clarify the directionality of the alcohol–POAG relationship in African populations [20, 41].

The unexpected inverse association between tranquillizer/painkiller use and POAG warrants careful interpretation. Participants who reported using tranquillizers showed 42% lower odds of POAG, whereas those who reported using painkillers showed 19% lower odds of POAG. These findings do not indicate a protective effect. However, they may reflect confounding by indication, where individuals with POAG may have different patterns of comorbidities compared with controls, a chance finding given the small sample size, or differential reporting bias, and controls who have other chronic conditions are more likely to report medication use. A more detailed assessment of specific medication classes, dosing patterns, and indications for use is necessary to accurately evaluate these relationships.

Marijuana use prevalence was low overall but significantly higher in the POAG group (1.8%) than in the controls (1.1%, *P* < 0.05). This crude association did not persist in the multivariable analysis, likely reflecting confounding by correlated substance use and sociodemographic variables rather than limited power. Reverse causation represents an alternative explanation as individuals with POAG may initiate marijuana use based on publicized claims of IOP reduction. Although acute cannabis exposure transiently lowers IOP (3–4 h) [42], this effect is clinically impractical for chronic disease management. Moreover, emerging data suggest that chronic cannabis use may adversely affect ocular perfusion and potentially increase the likelihood of POAG through pressure-independent mechanisms [43], contradicting its popular perception as a therapeutic agent.

The extremely low prevalence of other illicit substances (< 0.2%) precluded a meaningful analysis of their association with POAG, although this may reflect both the actual low prevalence and underreporting due to social desirability bias [44].

### Implications and limitations

Our findings have important implications for POAG prevention and management in African populations. The association between alcohol consumption and tobacco smoking observed in our study suggests that substance use counselling should be integrated into POAG care, with eye care providers routinely screening and providing appropriate cessation resources. If these observations are confirmed in well-designed and adequately powered confirmatory studies, public health messages about POAG prevention should include information about substance use as a risk factor, as most current campaigns in Africa focus primarily on regular examinations and family history [45]. Our findings also underscore the need for a more nuanced assessment of substance use patterns in POAG research, as simple binary classifications may obscure important relationships.

The strengths of this study include its large sample size of over 9,400 participants, inclusion of multiple substance use categories, and examination of an understudied African population.

However, this study had some limitations. The cross-sectional design precludes definitive conclusions regarding causality and temporal relationships. Reliance on self-reported substance use data is subject to recall and social desirability biases, potentially leading to underreporting and misclassification. Cumulative exposure metrics, including smoking pack-years and lifetime alcohol units, were not captured in this study, precluding a dose–response analysis. Future studies in this population should incorporate these measures to better characterize the relationship between cumulative tobacco and alcohol exposure and the risk of POAG. Finally, selection bias cannot be entirely ruled out, as our control group was mainly drawn from the same clinical settings.

## Conclusions

This study demonstrated significant associations between substance use patterns and POAG in the African population, with alcohol consumption and tobacco smoking showing particularly strong relationships with the disease. These findings suggest that substance use behaviors represent potentially modifiable risk factors, warranting attention in POAG prevention and management strategies. Given the substantial burden of POAG in Africa and the increasing prevalence of substance use in many African countries, these findings have important implications for public health policies and clinical practice. Future prospective studies are required to confirm these associations, establish temporal relationships, and evaluate whether substance use modifications can reduce the incidence of POAG or slow disease progression.

## Supplementary Information

Supplementary Material 1

## Data Availability

The data that support the findings of this study are available from the corresponding author upon reasonable request. Due to ethical and data protection considerations, individual-level data are not publicly available.

## Declarations

**Ethics approval and consent to participate**

The study protocol was reviewed and approved by the University of Ibadan/University College Hospital Ethics Review Committee (UI/EC/17/0394) and the relevant ethics committees of participating institutions, (The Ghana Health Service Review Committee: GHS-ERC 011/12/20, Korle Bu Teaching Hospital Review Committee: KBTH-IRB/00033/2021, University of Nigeria Teaching Hospital Ethics Review Committee: UNTH/CSA/329/OL.5 IRB00002323). Written consent to participate was obtained from all potential participants in their local language before screening and any study-related interventions (including examination for eligibility). Only eligible participants who consented to participate were enrolled in the study.
