# Supplementary material for: Association between alcohol and tobacco use and primary open-angle glaucoma: the Eyes of Africa study
Source: Eye Vis (Lond). 2026 Oct 10;13:44. doi: 10.1186/s40662-026-00515-y (PMC13653388; doi:10.1186/s40662-026-00515-y)
Supplement: Supplementary file 1 [file 40662_2026_515_MOESM1_ESM.docx]

**Supplementary Material**

**Supplementary Table 1.** Prevalence of substance consumption among individuals diagnosed with primary open-angle glaucoma (POAG) and those without the condition in Africa.

| **Variables** | **POAG (%)** | **Controls (%)** | **χ^2^** | ***P* value** |
| --- | --- | --- | --- | --- |
| Sedatives (n = 9232) |  |  | 1.64 | 0.21 |
| Never used | 3384 (66.3) | 2688 (65.1) |  |  |
| Ever used | 1717 (33.7) | 1443 (34.9) |  |  |
| Tranquillizers (n = 9197) |  |  | 17.39 | < 0.001* |
| Never used | 4951 (97.4) | 3941 (95.8) |  |  |
| Ever used | 133 (2.6) | 172 (4.2) |  |  |
| Painkillers (n = 9372) |  |  | 32.08 | < 0.001* |
| Never used | 1618 (31.3) | 1094 (26.0) |  |  |
| Ever used | 3546 (68.7) | 3114 (74.0) |  |  |
| Stimulants (n = 9318) |  |  | 2.27 | 0.13 |
| Never used | 4878 (94.8) | 3983 (95.5) |  |  |
| Ever used | 268 (5.2) | 189 (4.5) |  |  |
| Marijuana, Hash, HC, or Grass (n = 9335) |  |  | 8.00 | 0.005* |
| Never used | 5063 (98.2) | 4135 (98.9) |  |  |
| Ever used | 92 (1.8) | 45 (1.1) |  |  |
| **Cocaine (n = 9342) |  |  | 3.90 | 0.08 |
| Never used | 5150 (99.9) | 4184 (>99.9) |  |  |
| Ever used | 7 (0.1) | 1 (<0.1) |  |  |
| **Crack cocaine (n = 9339) |  |  | 0.77 | 0.47 |
| Never used | 5152 (99.9) | 4180 (>99.9) |  |  |
| Ever used | 5 (0.1) | 2 (<0.01) |  |  |
| **Hallucinogens (n = 9335) |  |  | 2.71 | 0.20 |
| Never used | 5146 (99.8) | 4179 (>99.9) |  |  |
| Ever used | 8 (0.2) | 2 (<0.1) |  |  |
| Inhalants (n = 9336) |  |  | 1.29 | 0.26 |
| Never used | 5127 (99.4) | 4160 (99.6) |  |  |
| Ever used | 31 (0.6) | 18 (0.4) |  |  |
| **Heroin (n = 9340) |  |  | 0.16 | 1.00 |
| Never used | 5155 (>99.9) | 4182 (>99.9) |  |  |
| Ever used | 2 (<0.1) | 1 (<0.1) |  |  |

HC = Hash Cannabis; *Statistically significant; ** Proportion of those who used the substance very low

**Supplementary Table 2.** Logistic regression of factors associated with primary open-angle glaucoma (BMI added).

| **Variable** | **AOR** | **95% CI** | ***P* value** |
| --- | --- | --- | --- |
| Sex |  |  |  |
| Female | Ref |  |  |
| Male | 1.44 | 1.19–1.74 | < 0.001* |
| Age (years) |  |  |  |
| 40–59 | Ref |  |  |
| 60–79 | 1.50 | 1.23–1.83 | < 0.001* |
| ≥ 80 | 2.27 | 1.79–2.87 | < 0.001* |
| Educational level |  |  |  |
| Less than secondary education | Ref |  |  |
| Secondary education and higher | 0.87 | 0.74–1.02 | 0.081 |
| Employment status |  |  |  |
| Employed | Ref |  |  |
| Unemployed | 1.66 | 1.34–1.98 | < 0.001* |
| Country of birth |  |  |  |
| Nigeria | Ref |  |  |
| Not in Nigeria | 0.52 | 0.40–0.68 | < 0.001* |
| Alcohol |  |  |  |
| Never drank | Ref |  |  |
| Former drinkers | 1.20 | 1.01–1.41 | 0.033* |
| Current drinkers | 1.87 | 0.58–6.07 | 0.297 |
| Tobacco |  |  |  |
| Never smoked | Ref |  |  |
| Former smokers | 1.40 | 1.08–1.81 | 0.011* |
| Current smokers | 1.68 | 1.23–2.31 | 0.001* |
| Tranquillizers |  |  |  |
| Never used | Ref |  |  |
| Ever used | 0.50 | 0.35–0.71 | < 0.001* |
| Painkillers |  |  |  |
| Never used | Ref |  |  |
| Ever used | 0.71 | 0.57–0.87 | 0.001* |
| Marijuana |  |  |  |
| Never used | Ref |  |  |
| Ever used | 1.19 | 0.74–1.91 | 0.41 |
| Alcohol use pattern |  |  |  |
| Not in the last 30 days | Ref |  |  |
| In the last 30 days | 1.29 | 1.13–1.47 | < 0.001* |
| Largest amount of drink ever drunk in a single day |  |  |  |
| ≤ 2 | Ref |  |  |
| > 2 | 1.13 | 0.93–1.37 | 0.210 |
| Obesity (BMI) |  |  |  |
| Normal Weight | Ref |  |  |
| Underweight | 0.31 | 0.21–0.46 | < 0.001* |
| Overweight | 0.89 | 0.74–1.08 | 0.234 |
| Obese | 0.83 | 0.67–1.04 | 0.103 |

AOR = adjusted odds ratio; CI = confidence interval; Ref = reference category; *Statistically significant
